# Effects of Transcutaneous Electrical Acupoint Stimulation on Systemic Inflammatory Response Syndrome of Patients after Percutaneous Nephrolithotomy: A Randomized Controlled Trial

**DOI:** 10.1155/2021/5909956

**Published:** 2021-08-12

**Authors:** Bin Que, Qing Tu, Jinlin Shi, Zhengzuo Wan, Yanan Li, Rong Zhou, Hong Yu, Jianhui Gan, Jianming Yu

**Affiliations:** ^1^Department of Anesthesiology, Hangzhou TCM Hospital Affiliated to Zhejiang Chinese Medical University, Hangzhou 310000, China; ^2^Department of Anesthesiology, Shanghai General Hospital, Shanghai Jiao Tong University School of Medicine, Shanghai 200000, China; ^3^Department of Anesthesiology, Tangshan People's Hospital, North China University of Science and Technology, Tangshan, Hebei 063000, China

## Abstract

**Purpose:**

Transcutaneous electrical acupoint stimulation (TEAS) is widely used. However, no study evaluated TEAS on systemic inflammatory response syndrome (SIRS) of patients after percutaneous nephrolithotomy (PCNL). The study was to evaluate TEAS on SIRS of patients after PCNL.

**Methods:**

67 patients were enrolled and divided into group TEAS and group sham TEAS. Data were collected from 60 participants finally. In the study, TEAS or sham TEAS on bilateral Shenshu (BL23), Yinlingquan (SP9), Hegu (LI4), and Neiguan (PC6) was performed continuously throughout the procedure. The primary outcome included the incidence of systemic inflammatory response syndrome (SIRS) within 48 h after surgery. The secondary outcomes included the serum levels of inflammatory cytokines, hemodynamics changes, complications, and hospital stay after surgery. The serum levels of tumor necrosis factor- (TNF-) *α* and interleukin- (IL-) 6, mean arterial pressure (MAP), and heart rate (HR) at 30 min before anesthesia (*T*_0_), the time after surgery (*T*_1_), 24 h postoperation (*T*_2_), and 48 h postoperation (*T*_3_) were recorded. The consumption of analgesic during surgery was also recorded, as well as the complications and duration of hospital stay after PCNL.

**Results:**

The incidence of SIRS in group TEAS was lower than group sham TEAS (30% vs. 6.67%, *p*=0.023). Compared with the sham TEAS group, both levels of TNF-*α* and IL-6 at *T*_1_, *T*_2_, and *T*_3_ were lower in the TEAS group (*p* < 0.05). The levels of MAP and HR in sham TEAS at *T*_1_, *T*_2_, and *T*_3_ were markedly higher than that in the TEAS group (*p* < 0.05). The total consumption of propofol and remifentanil during surgery in group TEAS was lower than that in the sham TEAS group. The incidence of hypotension, hypertension, emergence agitation, and postoperative nausea and vomiting (PONV) was also lower in group TEAS after PCNL (*p* < 0.05).

**Conclusions:**

TEAS could effectively reduce the incidence of SIRS and inflammatory cytokines for patients who underwent PCNL. In addition, TEAS helped to maintain the hemodynamic stability and cut down the consumption of analgesics during PCNL, reducing the complications after PCNL.

## 1. Introduction

Percutaneous nephrolithotomy (PCNL) has the advantages of less trauma and faster recovery, which gradually becomes the first-line treatment for upper urinary calculi removal [1]. With the improvement of surgical instruments and surgical techniques, the incidence of perioperative adverse events during PCNL was gradually decreasing. However, perioperative inflammatory response and even the systemic inflammatory response syndrome (SIRS) may occur after this procedure. A study reported the incidence of 27.6% fever after PCNL, 1.5–37% of whom developed SIRS [2, 3]. Although benefits provided, the procedure might spread the local urinary tract infections and pathogenic factors, which cause excessive activation of inflammatory cells, resulting in excessive release of various inflammatory cytokines and systemic hyperinflammatory reactions [4]. Transcutaneous electrical acupoint stimulation (TEAS) is a new emerging type of acupuncture combined with transcutaneous electrical nerve stimulation (TENS) and acupoint therapy [5]. A previous study had shown that TEAS could effectively reduce the consumption of remifentanil and the incidence of postoperative vertigo and itching after general anesthesia [6]. TEAS could also help to maintain the intraoperative hemodynamics during cardiac surgery [7]. In addition, acupuncture could reduce the release of inflammatory substances and produce anti-inflammatory effects via the action of specific acupuncture points [8, 9]. But the underlying mechanisms remained unclear. In science, PCNL often induces potent inflammatory response and some lethal complications, and effective measures should be applied to prevent it. Our study was to assess the effects of TEAS on the SIRS and levels of inflammatory cytokines of patients after PCNL.

## 2. Materials and Methods

### 2.1. Participants

The study was conducted in accordance with the Helsinki Declaration and the Good Clinical Practice Guidelines and approved by the Medical Ethics Committee of Tangshan People's Hospital, North China University of Technology (No. RMYY-YWLL-2017-1110). The study protocol was also registered (http://www.chictr.org.cn; identifier: ChiCTR1800018254). Informed consent was obtained from all participants in the study.

Participants who underwent PCNL were enrolled from September 2018 to December 2019 in the study. The eligibility criteria were as follows: (1) patients agreed and signed informed consent, (2) patients with normal cognitive function and skilled communication, (3) patients with American Society of Anesthesiology (ASA) physical status graded I–III, and (4) patients without serious organ or system diseases. Exclusion criteria were as follows: (1) patients allergic to the drugs and their related components in this study, (2) patients with pacemaker, (3) patients who have used painkillers or have a history of significant drug abuse, taking preoperative anti-inflammatory medications (e.g., nonsteroidal anti-inflammatory drugs (NSAIDs)) or steroids, (4) patients with a history of acupoint stimulation or acupuncture electrode treatment, (5) patients with acupuncture stimulation sites that are infected, having trauma, or not suitable for acupoint stimulation for other reasons, (6) patients who have a history of uncontrolled hypertension, diabetes, and malignant tumor, (7) patients who are pregnant or have childbearing potential, (8) patients who participated in other clinical trials, and (9) patients with bilateral renal calculi.

### 2.2. Randomization and Blinding

This study was a prospective, randomized, controlled trial. The perioperative management sequences of TEAS (group TEAS) and sham TEAS (group sham TEAS) were determined, and randomization was stratified according to the time of admission in a block size of 10. Eligible participants were randomly assigned to receive either TEAS or sham TEAS via a central randomization system for clinical research using a 1 : 1 ratio. The random number list was generated by an independent statistician, and block size was disclosed to other researchers. An independent, blinded statistician concealed the file of the generated random number table using a password and provided information regarding which group the participant assigned. Researchers who measured outcomes and researchers who performed data management and statistical analysis were blinded to each participant's allocation status. The reviewer responsible for the statistical results was not allowed to engage in any dialogue with the participants. Practitioners were not involved in measuring treatment outcomes or data analysis. In addition, the patients involved in the study were treated separately to avoid mutual communication. Participants were allowed to withdraw from the trial at any time.

### 2.3. Intervention and Anesthesia Protocols

**Figure 1:**
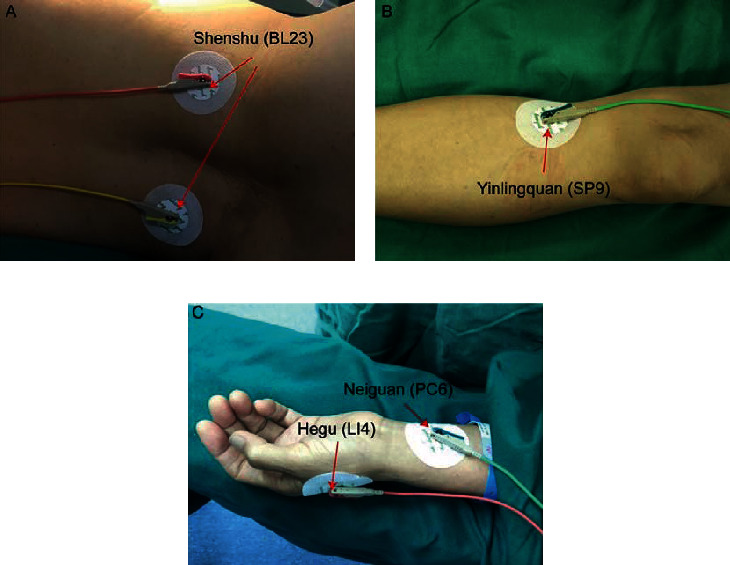
Depiction of the target acupoints. (a) BL23, Shenshu, located about 5 cm outside the spinous process of the second lumbar vertebra. (b) SP9, Yinlingquan, located at the beginning of the soleus muscle, between the posterior tibia border and gastrocnemius muscle. (c) LI4, Hegu, localized with the hand in flat position and with the thumb and index finger spread, in the slight depression between the 1st and 2nd metacarpal bones; PC6, Neiguan, localized with the forearm extended and the palm faced upwards, in the center of the palmar aspect of the forearm, between the tendons of the long palmar muscle and the radial flexor muscle of the wrist.

The procedure of TEAS was performed by an independent anesthetist (a sealed envelope containing the grouping information of the participant was sent to the anesthetist before anesthesia), who was not allowed to participate in the process of data collection and analysis. For participants in group TEAS, TEAS was implemented on bilateral Shenshu (BL23, located about 5 cm outside the spinous process of the second lumbar vertebra), Yinlingquan (SP9, located at the beginning of the soleus muscle, between the posterior tibia border and gastrocnemius muscle), Hegu (LI4, localized with the hand in flat position and with the thumb and index finger spread, in the slight depression between the 1st and 2nd metacarpal bones), and Neiguan (PC6, localized with the forearm extended and the palm faced upwards, in the center of the palmar aspect of the forearm, between the tendons of the long palmar muscle and the radial flexor muscle of the wrist) 30 min before the induction of anesthesia (Figure 1). After skin disinfection, four pairs of electrodes were, respectively, pasted on the target acupoints and connected with the HANS 200A acupoint stimulator (Nanjing Jisheng Medical Technology Co., Ltd., China). The frequency was set as 2/100 Hz in the dense-disperse mode (in which the frequency was automatically alternated at every 3 s between 2 and 100 Hz). The current intensity was 5–30 mA (5–10 mA for the upper limbs, and 10–30 mA for the lower limbs and trunk) [10], and the intensity of TEAS was adjusted to a tolerable level. TEAS onsets from 30 min before anesthesia induction till the end of the surgery (at the intervals of 30 min). For participants in group sham TEAS, the electrodes were also pasted on the same target acupoints, but the TEAS was not operated. All patients were informed that TEAS worked throughout the surgery. The electrodes were well protected from detaching during the operation.

A standard anesthetic protocol was followed. The surgeries were performed by a same surgical team under general anesthesia. Upon entering the operation room, a dedicated intravenous cannula was inserted by circuit nurse. The noninvasive blood pressure (NIBP), heart rate (HR), electrocardiographic monitoring (ECG), blood oxygen saturation (SpO_2_), and bispectral index (BIS) were continuously monitored during the surgery. Anesthesia induction was performed by using fentanyl 2-3 *μ*g kg^−1^ i.v., propofol 2–6 mg kg^−1^ i.v., and rocuronium 0.5 mg kg^−1^ i.v. During the period of induction and maintenance of general anesthesia, propofol 6–12 mg kg^−1^ h^−1^ and remifentanil 0.2–0.6 *μ*g kg^−1^ min^−1^ were continuously infused and controlled by the same closed-loop automatic control system. Additional rocuronium was titrated to maintain an adequate level of muscle relaxation. The BIS value was maintained between 40 and 50 till the end of the surgery. Dezocine 5 mg and palonosetron 0.25 mg were administrated to control the postoperative pain and prevent postoperative nausea and vomiting (PONV) for all patients. All anesthetics were discontinued at the end of the surgery. All participants were transferred to the postanesthesia care unit (PACU) after extubation and escorted back to the ward after monitoring for 60 min.

### 2.4. Outcomes

The primary outcome included the incidence of SIRS after PCNL. SIRS was diagnosed when patients met two or more of the following criteria [11]: (a) body temperature lower than 36°C or higher than 38°C, (b) HR greater than 90 beats/min, (c) respiratory rate greater than 20 breaths/min or partial pressure of carbon dioxide in artery (PaCO_2_) less than 4.3 kPa (≈32 mmHg), and (d) white blood cell count (WBC) greater than 12^*∗*^10^9^/*L* or less than 4^*∗*^10^9^/L. SIRS was diagnosed and recorded by a senior anesthesiologist during the follow-up (48 h after PCNL), who was blinded to the grouping information. The incidence of SIRS was recorded within 48 h after PCNL.

The secondary outcomes included the serum levels of inflammatory cytokines (TNF-*α* and IL-6), MAP and HR, consumption of anesthetics, and incidence of other complications after PCNL. Peripheral blood samples were taken for detecting the serum cytokines of TNF-*α* and IL-6 at 30 min before anesthesia (*T*_0_), the time after surgery (*T*_1_), 24 h postoperation (*T*_2_), and 48 h postoperation (*T*_3_). Blood samples were drawn using the heparin-containing syringe and then centrifuged at 3000 g for 15 min. The serum was extracted and stored in the freezer at −80°C for further assay. The serum levels of TNF-*α* and IL-6 were measured by the enzyme-linked immunosorbent serologic assay (ELISA) method (Lianke Biotech Co., Ltd., Hangzhou, China) according to manufacturer's instructions.

The MAP and HR at *T*_1_, *T*_1_, *T*_2_, and *T*_3_, consumption of anesthetics during the surgery, incidences of other complications, and duration of hospital stay postoperation were also recorded.

### 2.5. Statistics Analysis

The sample size was calculated by using a webtool (https://sample-size.net) based on a previous meta-analysis [12], which assessed the effects of TEAS on PONV after surgery. A sample size of 22 participants for each group was sufficient to detect a significant difference (*α* = 5%) with a statistical power (*β* value) of 0.8. We presumed a 20% drop-off rate, indicating a sample size of 27 for each group.

Data are presented as the means and standard deviations (SD) for continuous variables and as proportions for categorical variables. Normally distributed continuous data (determined by the Kolmogorov–Smirnov method) were compared using Student's *t*-test. The chi-square test was used to analyze categorical variables. One-way ANOVA was used to analyze differences between the baseline values and other time points. Repeated measurements were used to analyze differences in the interaction effects between groups and different time points. A two-tailed *p* value of <0.05 was considered statistically significant. Data were processed via using SPSS version 18.0 (SPSS Inc., Chicago, IL, USA).

## 3. Results

### 3.1. Demographics and Perioperative Characteristics

**Figure 2:**
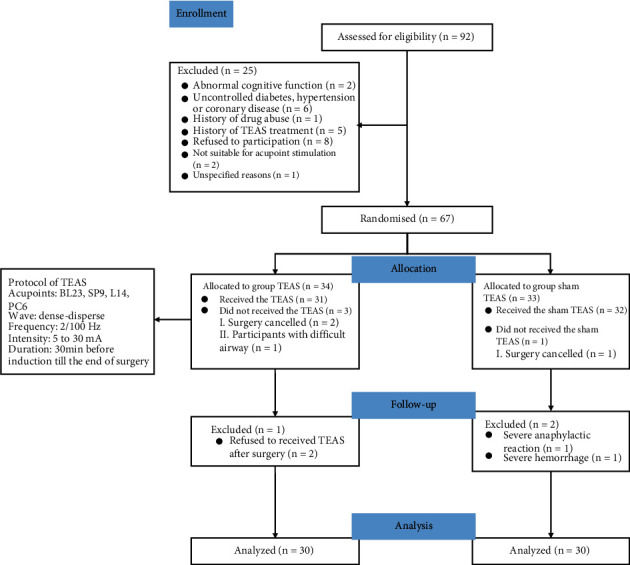
Flow of participants randomized to receive TEAS or sham TEAS. TEAS, transcutaneous electrical acupoint stimulation.

We finally enrolled 67 participants in the study. Throughout the study, there were 2 surgeries cancelled in group TEAS, as well as 1 in group sham TEAS. One patient in group TEAS has potential difficult airway. One patient refused to receive TEAS for intolerance of electrical stimulation in group TEAS and two patients had severe complications during surgery. Finally, available data were obtained from 60 patients (Figure 2). There were no significant differences in the baseline characteristics among two groups (Table 1).

### 3.2. Incidence of SIRS in PACU

Nine patients (30%) developed SIRS after PCNL in group sham TEAS, which was similar to a previous study [13], while two patients (6.67%) developed SIRS in the TEAS group (Table 2), which was lower than that in the sham TEAS group (*p* < 0.05).

### 3.3. Peripheral Serum Levels of TNF-*α* and IL-6

**Figure 3:**
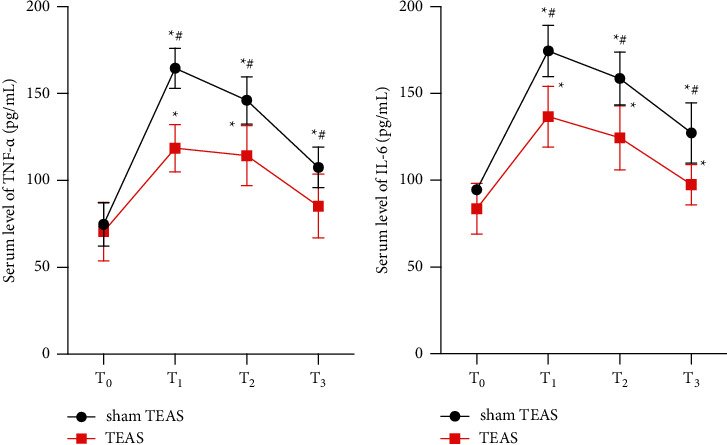
(a) Serum levels of TNF-*α* after surgery between groups. (b) Serum levels of IL-6 after surgery between groups. Values are presented as mean ± standard (SD). TEAS, transcutaneous electrical acupoint stimulation. *T*_0_, 30 min preoperation; *T*_1_, time after operation; *T*_2_, 24 h postoperation; *T*_3_, 48 h postoperation; TNF, tumor necrosis factor; IL, interleukin. ^*∗*^*P* < 0.5 vs. *T*_0_. ^#^*P* < 0.5 vs. group sham TEAS.

Compared with the baseline level before anesthesia, the levels of TNF-*α* and IL-6 at *T*_1_, *T*_2_, and *T*_3_ were significantly increased in the sham TEAS group (*P* < 0.05), while in the TEAS group, levels of TNF-*α* at *T*_1_, *T*_2_ and IL-6 at *T*_1_, *T*_2_, and *T*_3_ were higher (*p* < 0.05). Compared with the sham TEAS group, both levels of TNF-*α* and IL-6 at *T*_1_, *T*_2_, and *T*_3_ were lower in the TEAS group (*p* < 0.05), indicating that TEAS inhibited the release of cytokines after PCNL (Figure 3).

### 3.4. Hemodynamic Changes

**Figure 4:**
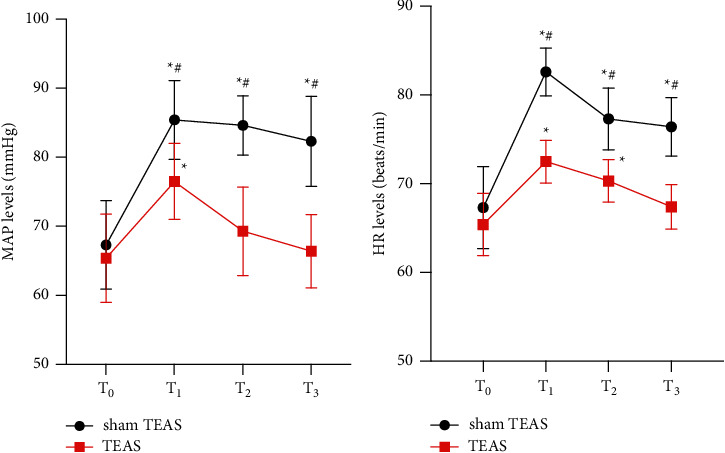
(a) MAP levels between groups. (b) HR levels between groups. Values are presented as mean ± standard (SD). TEAS, transcutaneous electrical acupoint stimulation. MAP, mean arterial pressure, HR, heart rate. ^*∗*^*P* < 0.5 vs. *T*_0_. ^#^*P* < 0.5 vs. group sham TEAS.

The basal MAP and HR levels of the two groups were similar (*p* > 0.05). Compared with *T*_0_, the levels of MAP and HR at *T*_1_, *T*_2_, and *T*_3_ were increased in the sham TEAS group, while MAP at *T*_1_ and HR at *T*_1_ and *T*_2_ were higher at the TEAS group (*p* < 0.05). However, the levels of MAP and HR in sham TEAS at *T*_1_, *T*_2_, and *T*_3_ were markedly higher than that in the TEAS group (*P* < 0.05), suggesting TEAS contributed to make a more stable hemodynamic change during the perioperative period (Figure 4).

### 3.5. Anesthetics Consumption, Complications, and Hospital Stay

The total consumption during surgery of propofol (*p*=0.016) and remifentanil (*p*=0.023) in group TEAS was lower than that in the sham TEAS group, the incidence of hypotension (*p*=0.042), hypertension (*p*=0.012), emergence agitation (*p*=0.018), and PONV (*p*=0.022). But the duration of hospital stay was similar between two groups (*p* > 0.05) Table 2.

## 4. Discussion

Nephrolithiasis is a very common disease in urological daily practice [14]. Nephrolithiasis is often accompanied by urinary tract infection. However, the surgical interventions may further amplify the infection and inflammatory response, resulting in drastic hemodynamic changes and other adverse events, such as hypertension, tachycardia [15], or even SIRS. TEAS was noninvasive, with similar effects of acupuncture and percutaneous electrical stimulation. A recent clinical study suggested that TEAS downregulates proinflammatory factors, such as C-X-C motif chemokine ligand 8 (CXCL8), IL-1, IL-6, TNF-*α*, and C–C motif chemokine ligand 2 (CCL2) in response to lower limb ischemia-reperfusion [16]. While, the animal study suggested that TENS inhibited the expression of IL-1*β*, TNF-*α*, and inducible nitric oxide synthase (iNOS) [17]. As a type of acupuncture, TEAS combines TENS and acupoint therapy together [5]. TEAS also helps to regulate the autonomic nervous system, contribute to reduce the sympathetic excitability and produced sedation effect [18], and stabilize hemodynamic changes [19, 20]. Clinical studies have found that acupuncture on SP9 relieved pain symptoms in most patients with renal colic [21]. According to the theory of traditional Chinese medicine (TCM), acupuncture on BL23 contributes to reduce the pressure in the renal pelvis, relax the smooth muscle of the ureter, and reduce the local edema caused by ureteral stones. Our previous study suggested that TEAS on BL23 and SP9 helped to alleviate postoperative pain for patients after ureteroscopic lithotripsy [22]. In fact, inflammation response is one of the common causes for postoperative pain. Studies showed that electrical stimulation of LI4 and PC6 acupoints inhibits sympathetic activation, enhances vagal activity, helps to maintain the hemodynamic stability, and reduces the stress response in vivo [23, 24]. Therefore, BL23, SP9, LI4, and PC6 were selected as the target acupoints in the study.

As a mini-invasive surgery, PCNL, it is still accompanied by various complications, including bleeding, infection, water poisoning, and extravasation. Surgery spreads the primary urinary tract infection, causing excessive release of a large number of inflammatory cytokines (such as IL-6, IL-8, and TNF-*α*). Serious infection may develop SIRS and eventually result in severe septic shock or even death. The peripheral level of IL-6 sensitively reflects the extent of tissue damage [25]. TNF-*α* elevates in the systemic inflammatory response, related to the severity of the disease. Elevated TNF-*α* initiates the NF-*κ*B signaling pathway, leading to a cascade of inflammatory cytokines release, which in turn enlarges the inflammatory effect [26]. Inflammatory response or even SIRS may easily occur during the procedure of PCNL. However, whether TEAS reduced the incidence of SIRS and inflammatory cytokines during PCNL was unknown. In our study, the incidence of SIRS in group sham TEAS was similar to a previous study [13]. Interestingly, the incidence of SIRS in group TEAS was lower than that in the TEAS group. We also tested the cytokines (TNF-*α* and IL-6) in peripheral blood. In line with the incidence of SIRS, the serum levels of TNF-*α* and IL-6 were also lower in the TEAS group than that in the sham TEAS group, indicating TEAS helped to inhibit the release of cytokines, thus reducing the incidence of SIRS after PCNL.

TEAS may also stabilize hemodynamic changes via regulating the autonomic nervous system and sedation effects [18–20]. In our study, hemodynamic changes were more stable in the TEAS group. In addition, the incidence of hypotension and hypertension in the TEAS group was also lower than that in the sham TEAS group. This may contribute to the effects of TEAS in regulating the autonomic nervous system [27].

Analgesic effects of TEAS were justified by many studies; it may be closely related to the activation of the endogenous analgesic system. Animal experiments suggested electroacupuncture exerted analgesic effects by affecting the release and response of neurotransmitters such as dopamine, serotonin, and acetylcholine [28, 29]. Studies also demonstrated that TEAS could directly inhibit the neural interactions associated with opioid analgesia in the spinal cord and increase the release of endogenous opioid peptides through central levels [30, 31]. In our study, TEAS reduced the consumption of propofol and remifentanil during surgery. Research suggested that electroacupuncture on LI4 contributed to sedation effects [32]. In our study, we also found TEAS reduced the incidence of emergence agitation in postanesthesia care unit. Studies have shown that electrical stimulation on PC6 could reduce the incidence of PONV [33]. The incidence of PONV in group TEAS was also lower than that in group sham TEAS. This may contribute to the inhibitory effects on vomiting center. In TEAS, the transmission of serotonin-3 (HT-3) is via activated adrenergic receptors and noradrenergic fibers [12].

Although, our study was positive of TEAS in reducing the incidence of SIRS, inflammatory response, and adverse events for patients after PCNL. There also existed several limitations in our study. First, since all the patients were comprised of the same hospital, more clinical research studies from the multicenter should be conducted to generalize the results of our study. Second, we only tested TNF-*α* and IL-6 in the study, whether TEAS which inhibited other cytokines still needs more study to justify it. Since there were no unified standards to set the mode, density, and duration of TEAS, we only set the TEAS according to the experience in our center. A recent study suggested that the acupoints, stimulation intensity, and the duration may together affect the effects of electroacupuncture [27]. Therefore, it still needs more studies to unify the standards of the clinical application of TEAS.

## 5. Conclusion

Our study reported that the application of TEAS could effectively reduce the incidence of SIRS and inflammatory cytokines of TNF-*α* and IL-6 for patients who underwent PCNL. In addition, TEAS also helped to maintain the hemodynamic stability, cut down the consumption of analgesics during PCNL, and reduce the adverse events after PCNL.

## Figures and Tables

**Table 1:** Baseline characteristics of participants.

Characteristics	Sham TEAS (*n* = 30)	TEAS (*n* = 30)	*P* value
Age (year, mean ± SD)	47.1 ± 16.3	45.4 ± 18.7	0.052

Sex (*n* (%))			
Male	18 (60)	19 (63.3)	0.629
Female	12 (40)	11 (36.7)	

Weight (kg, mean ± SD)	57.6 ± 13.2	58.4 ± 14.8	0.828

ASA physical status (*n* (%))			
I	6 (20)	7 (23.3)	—
II	16 (53.3)	14 (46.7)	—
III	8 (26.7)	9 (30)	—

Stone position (*n* (%))			
Left	12 (40)	19 (63.3)	0.240
Right	18 (60)	11 (36.7)	
Stone (multiple)	23 (83.3)	20 (66.7)	

Staghorn calculi (*n* (%))	17 (56.7)	21 (70)	0.506
Diabetes (*n* (%))	12 (40)	9 (30)	0.408
Hypertension (*n* (%))	7 (23.3)	6 (20)	0.712

Preoperative urine culture (*n* (%))			
Positive	12 (40)	10 (33.3)	0.579
Negative	18 (60)	20 (66.7)	

Temperature (°C, mean ± SD)	36.2 ± 1.2	36.4 ± 0.9	0.135
White cell count (×10^9^/L)	7.14 ± 1.84	7.23 ± 1.77	0.374
Hemoglobin (g/dL)	12.32 ± 2.43	12.83 ± 1.04	0.481
Urea nitrogen (mmol/L)	5.82 ± 0.64	5.37 ± 0.82	0.288
Serum creatinine (*μ*mol/L)	90.37 ± 3.63	85.66 ± 4.21	0.736

Tract size			
18–20 F	4 (13.3)	7 (23.3)	0.205
21–24 F	10 (33.3)	8 (26.7)	
25–30 F	16 (53.3)	15 (50)	

Volume of transfusion (mL, mean ± SD)	743.5 ± 76.2	764.7 ± 82.5	0.536
Operation time (min, mean ± SD)	114.2 ± 10.7	110.4 ± 12.8	0.278
Anesthesia time (min, mean ± SD)	138.2 ± 20.2	132.7 ± 18.7	0.473

*Note.* Values are presented as mean mean ± SD or *n* (%). TEAS, transcutaneous electrical acupoint stimulation; SD, standard deviation; ASA, American Society of Anesthesiologists.

**Table 2:** Anesthetic consumption and complications after surgery.

Variables	Sham TEAS (*n* = 30)	TEAS (*n* = 30)	*P* value
SIRS (*n* (%))	9 (30)	2 (6.67)	0.023^#^
Propofol (mg, mean ± SD)	625.7 ± 261.4	469.2 ± 181.7	0.016^#^
Remifentanil (*μ*g, mean ± SD)	1107.5 ± 327.4	878.7 ± 177.4	0.023^#^
Volume of rehydration (min, mean ± SD)	234.2 ± 73.3	228.5 ± 81.2	0.734
Hypothermia (*n* (%))	17 (32.7)	15 (27.8)	0.582
Hyperthermia (*n* (%))	8 (15.4)	8 (14.8)	0.935
Hypotension (*n* (%))	14 (26.9)	6 (11.1)	0.042^#^
Hypertension (*n* (%))	15 (28.8)	5 (9.3)	0.012^#^
Emergence agitation (*n* (%))	14 (26.9)	5 (9.3)	0.018^#^
PONV (*n* (%))	15 (28.8)	6 (11.1)	0.022^#^
Vertigo (*n* (%))	13 (25)	10 (18.5)	0.418
Sleepiness (*n* (%))	14 (26.7)	9 (16.7)	0.200
Skin itch (*n* (%))	7 (13.5)	5 (9.3)	0.495
Remedy analgesic (mg, mean ± SD)	142.5 ± 23.6	87.5 ± 10.2	0.028^#^
Hospital stay (days, mean ± SD)	6.43 ± 2.15	5.31 ± 1.13	0.072

*Note.* Values are presented as mean ± standard deviation (SD) or *n* (%). TEAS, transcutaneous electrical acupoint stimulation; PACU, postanesthesia care unit; PONV, nausea and vomiting. ^#^*P* < 0.05, compared with group sham TEAS.

## Data Availability

The data of the study are available from the corresponding author, Jianming Yu (sjlints@163.com).
